# *Antrodia camphorata* Increases Insulin Secretion and Protects from Apoptosis in MIN6 Cells

**DOI:** 10.3389/fphar.2016.00067

**Published:** 2016-03-21

**Authors:** Chi Teng Vong, Hisa Hui Ling Tseng, Yiu Wa Kwan, Simon Ming-Yuen Lee, Maggie Pui Man Hoi

**Affiliations:** ^1^State Key Laboratory of Quality Research in Chinese Medicine, Institute of Chinese Medical Sciences, University of MacauMacau, China; ^2^School of Biomedical Sciences, Faculty of Medicine, The Chinese University of Hong KongHong Kong, China

**Keywords:** *Antrodia camphorata*, insulin secretion, pancreatic β-cell death, PPAR-γ, type 2 diabetes mellitus

## Abstract

*Antrodia camphorata* is a Taiwanese-specific fungus which has been used clinically to treat hypertension, immune- and liver-related diseases and cancer; however, it has never been studied in type 2 diabetes mellitus (T2DM). Hyperglycemia in T2DM causes endoplasmic reticulum (ER) stress, leading to β-cell dysfunction. During chronic ER stress, misfolded proteins accumulate and initiate β-cell apoptosis. Moreover, β-cell dysfunction leads to defect in insulin secretion, which is the key process in the development and progression of T2DM. Therefore, the aim of the present study was to examine the effects of *A. camphorata* on insulin secretion and ER stress-induced apoptosis in a mouse β-cell line, MIN6, and their underlying mechanisms. We demonstrated that the ethanolic extract of *A. camphorata* increased glucose-induced insulin secretion dose-dependently through peroxisome proliferator-activated receptor-γ (PPAR-γ) pathway, and upregulated genes that were involved in insulin secretion, including PPAR-γ, glucose transporter-2 and glucokinase. Furthermore, *A. camphorata* slightly increased cell proliferation, as well as protected from ER stress-induced apoptosis in MIN6 cells. In conclusion, this study provided evidences that *A. camphorata* might have anti-diabetic effects and could be a novel drug for T2DM.

## Introduction

*Antrodia camphorata* is a Taiwanese-specific fungus, which has been used as traditional medicine to treat cancer, hypertension and skin itching, and it also has anti-oxidant and anti-inflammatory effects (Geethangili and Tzeng, 2011). Previous study demonstrated that the aqueous extract of *A. camphorata* exhibited hypolipidemic activity and reduced plasma triglyceride levels in hypercholesterolemic rats (Suk et al., 2008). Other studies also showed that *A. camphorata* ameliorated hepatic steatosis, hyperlipidemia and metabolic syndrome in high-fat-diet mice (Chou et al., 2013; Kuo et al., 2015).

Recently, it has been suggested that *A. camphorata* might contain PPAR-γ ligands, which resulted in hypotriglyceride effects (Suk et al., 2008). PPAR-γ belongs to the nuclear receptor superfamily which is also expressed in β-cells, and it forms heterodimers with RXR in order to bind to the PPRE on gene promoters and activates gene transcription (Kim and Ahn, 2004). It has been reported the presence of PPRE in the GLUT-2 and GLK promoters, which are involved in insulin secretion (Kim et al., 2000, 2002). Several studies have demonstrated that PPAR-γ agonists could increase glucose-induced insulin secretion in primary islets and β-cell lines (Ishida et al., 2004; Kim et al., 2008; Evans-Molina et al., 2009). However, the effect of *A. camphorata* in insulin secretion has not been studied in pancreatic β-cells.

β-cell dysfunction leads to defect in insulin secretion, which is the key process in the development and progression of T2DM. In T2DM, chronic insulin resistance and a progressive decline in β-cell function result in β-cell apoptosis and dysfunction (Butler et al., 2003). Therefore, it has been suggested that preserving β-cell function and mass is an important therapeutical target for the treatment of T2DM (Buchanan et al., 2002; Del Prato et al., 2007; Vetere et al., 2014). ER stress is one of the main causes of β-cell apoptosis and dysfunction in T2DM (Butler et al., 2003; Prentki and Nolan, 2006; Eizirik et al., 2008). It is the accumulation of misfolded proteins in the ER, which activates UPR (Leibowitz et al., 2010), and the UPR prevents the accumulation of misfolded protein in the cells. The UPR is regulated by different proteins localized to the ER membrane: protein kinase R (PKR)-like ER kinase (PERK), IRE1α and activating transcription factor 6α (ATF6α; Leibowitz et al., 2010; Back et al., 2012). During chronic ER stress, these pathways fail to degrade misfolded proteins, which initiate β-cell apoptosis (Rutkowski et al., 2006).

In this study, we investigated the effects of *A. camphorata* on insulin secretion and ER stress-induced apoptosis in a β-cell line, MIN6. Our results showed that *A. camphorata* increased insulin secretion in a dose-dependent manner through PPAR-γ, and it also protected the cells from ER stress-induced apoptosis.

## Materials and Methods

### Materials

The ethanolic extract of *A. camphorata* was provided by Biotech Lantyng Company (Taipei, Taiwan). Cell culture reagents were obtained from Gibco (USA). GW9662, Rosaglitazone, and MTT were purchased from Sigma (USA). PPAR-γ (D69), ki-67 (D3B5) mAb, Caspase-3, β-actin, α,β-Tubulin and GAPDH antibodies, and anti-rabbit-HRP secondary antibodies were obtained from Cell Signaling Technology (USA), while GLK, p-IRE1α (phospho-S724), IRE1α antibodies were obtained from Abcam (USA). p-PERK (phosphor-Thr981), PERK (aa947-996) antibodies were purchased from LifeSpan BioSciences (USA), while GLUT-2 (H-67) and ATF6α (H-280) antibodies were purchased from Santa Cruz Biotechnology (USA).

### Cell Culture

MIN6 cells were cultured with DMEM highglucose (25 mM) supplemented with 10% fetal bovine serum and 1% penicillin–streptomycin, and equilibrated with 5% CO_2_ and 95% air at 37°C. The experiments were performed between passages 16 and 24.

### Extraction of *A. camphorata*

The mycelia of *A. camphorata* were cultured in 1 L of growth medium containing 0.1 g NaCl, 10 g peptone, 2 g yeast extract, 10 g agar, and 10 g cereal mixture (rice, wheat, and corn), at pH 7.5 and 25°C for 12–14 weeks. 1 kg of *A. camphorata* mycelia were extracted twice with a 10-fold ethanol solution to obtain two ethanolic extracts by ultrasound-assisted extraction method at 50°C. The ethanolic extracts were then concentrated to yield 230 g crude extract.

### Glucose-Stimulated Insulin Secretion

The cells were cultured in 24-wells plates with high glucose DMEM (25 mM), and were treated with *A. camphorata* (0–500 ng/ml) for 24 h. GW9662 (50 μM, Tocris Bioscience, USA), a PPAR-γ inhibitor, was co-treated with *A. camphorata* for 24 h, and Rosglitazone (50 μM, Sigma–Aldrich, USA), a PPAR-γ agonist, was used as a positive control. After 24 h of treatment, the cells were washed twice with Krebs–Ringer bicarbonate buffer (KRBB: CaCl_2_ 2.5 mM; KCl: 4.7 mM; KH_2_PO_4_: 1.2 mM; MgCl_2_: 1.2 mM; NaCl: 120 mM; HEPES: 10 mM; NaHCO_3_: 25 mM; and pH = 7.4) no glucose, and incubated with KRBB 3 mM glucose for 30 min. The cells were washed twice with KRBB no glucose before incubating with KRBB 5.5 or 16.7 mM glucose for 1 h. The supernatants were collected and insulin was measured by mouse insulin ELISA (Mercodia, USA).

### MTT Assay

MTT assay was used to determine cell viability, and TG was used as an ER stress inducer. The cells were pre-treated with *A. camphorata* (0–500 ng/ml) for 24 h, and then were treated with 1 μM TG for 24 h to induce cell death. MTT (1 mg/ml) was added to the cells, and was incubated for 4 h. The absorbance was measured at 570 nm.

### Apoptosis Assay

Annexin V–PI staining was used to measure cell apoptosis by flow cytometry. The cells were incubated with *A. camphorata* (0–500 ng/ml) for 24 h, and then were stimulated with 1 μM TG for 24 h. The staining was performed according to the manufacturer’s protocol (BD Biosciences, USA). Annexin V–fluorescein isothiocyanate (FITC) positive cells were defined as early apoptotic cells, whereas Annexin V and PI positive cells were defined as late apoptotic cells. Analyses were performed using FlowJo 7.6.1 software.

### Ki-67 Staining

The cells were cultured in 96-wells plates and were treated with 50–500 ng/ml *A. camphorata* for 24 h. The cells were then fixed with fixative for 15 min and permeabilized with 0.5% Triton X-100 for 10 min. The cells were blocked with 20% donkey serum for 30 min. Subsequently, the cells were stained with ki-67 (D3B5) monoclonal antibody (Alexa Fluor 488 conjugate) for 1 h and DAPI (1 μg/ml) for 10 min. Images were taken with 20× magnification by IN Cell Analyser 2000 Imaging System (GE Healthcare Life Sciences, USA), and were analyzed with Image J software.

### Western Blot Analysis

The cells were either treated with *A. camphorata* (0–500 ng/ml) for 24 h only, or were then treated with 1 μM TG for 6 or 24 h. After treatment, the protein was extracted with ice-cold lysis buffer, and nuclear proteins were extracted using the Nuclear and Cytoplasmic Extraction Kit (Pierce, France) for detection of the PPAR-γ antibody only. The protein concentrations of the lysates were measured by the bicinchoninic acid kit (Pierce, France). 40–60 μg proteins were used and separated by 8–10% SDS-PAGE gels, and were then transferred onto the nitrocellulose membranes. Membranes were incubated with PPAR-γ, GLUT-2, GLK, Caspase-3, p-IRE1α, IRE1α, p-PERK, PERK, ATF6α antibodies and anti-rabbit-HRP secondary antibodies, and blots were developed by enhanced chemiluminescence (GE Healthcare Life Sciences, USA) with an imaging system (Bio-Rad Laboratories, USA). GAPDH, β-actin, and α,β-tubulin were used as housekeeping controls.

### Real-Time PCR Analysis

The cells were treated with *A. camphorata* (0–500 ng/ml) for 24 h. After treatment, total RNA was extracted using RNeasy Mini Kit (Qiagen, USA), and cDNA was synthesized by reverse transcription. cDNA was quantified using Sybr Green assays by ViiA 7 Real-Time PCR System (Applied Biosystems, USA). The primers used were as follows: GLUT-2 (forward: 5′-TCAGAAGACAAGATCACCGGA-3′; reverse: 5′-GCTGGT GTGACTGTAAGTGGG-3′), GLK (forward: 5′-TGAGCCG GATGCAGAAGGA-3′; reverse: 5′-GCAACATCTTTACACT GGCCT-3′), β-actin (forward: 5′-GGCTGTATTCCCCT CATCG-3′; reverse: 5′-CCAGTTGGTAACAATGCCATGT-3′). β-actin was used as an internal control. Gene expressions were calculated using the ΔΔ*C*_t_ method, and were normalized to control.

### Statistical Analysis

The results were expressed as mean ± SEM (standard error of the mean). Statistical significance was determined by *t*-test or one-way ANOVA followed by Dunnett’s test, using GraphPad Prism 5.0. *P <* 0.05 was considered as significant.

## Results

### *Antrodia camphorata* Enhanced Glucose-Induced Insulin Secretion through PPAR-γ Pathway

**FIGURE 1:**
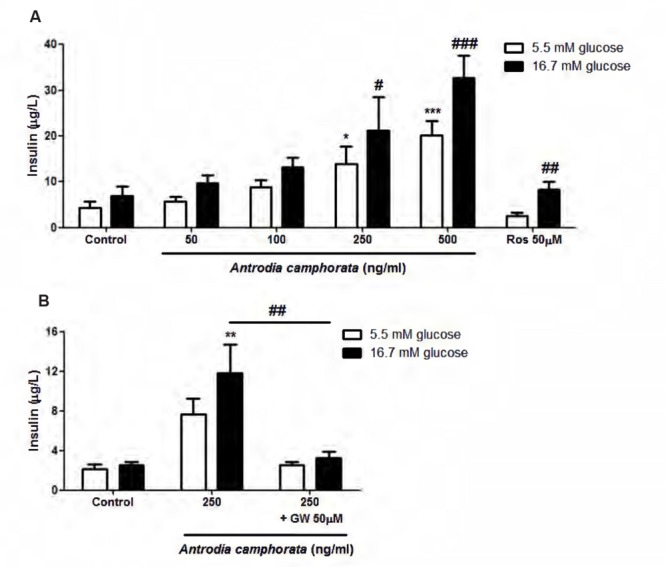
***Antrodia camphorata* increased insulin secretion through PPAR-γ in MIN6 cells.** The cells were treated with *A. camphorata* for 24 h, and were stimulated with Krebs–Ringer bicarbonate buffer (KRBB) 5.5 mM or 16.7 mM glucose for 1 h. **(A)** Insulin levels were determined using a mouse insulin ELISA kit. 50 μM Rosglitazone (Ros), a PPAR-γ agonist, was used as a positive control, *n* = 4–6. One-way ANOVA; *^∗^P* < 0.05, *^∗∗∗^P* < 0.001 vs. control with KRBB 5.5 mM glucose; *^#^P* < 0.05, *^##^P* < 0.01, *^###^P* < 0.001 vs. control with KRBB 16.7 mM glucose. **(B)** The cells were treated with 250 ng/ml *A. camphorata* in the absence and presence of 50 μM GW9662 (GW), PPAR-γ inhibitor, *n* = 5. *^∗∗^P* < 0.01 vs. control with KRBB 16.7 mM glucose; *^##^P* < 0.01. Results were expressed as mean ± SEM.

Firstly, we investigated the effect of the ethanolic extracts of *A. camphorata* on glucose-induced insulin secretion in MIN6 cells. After treatment with *A. camphorata* for 24 h, MIN6 cells were incubated with KRBB 5.5 mM or 16.7 mM glucose for 1 h, the supernatants were then collected for insulin ELISA. *A. camphorata* (50–500 ng/ml) enhanced glucose-induced insulin secretion dose-dependently in MIN6 cells (**Figure 1A**). Rosglitazone, a PPAR-γ agonist, was used as a positive control. 50 μM Rosglitazone significantly increased insulin secretion at 16.7 mM glucose (**Figure 1A**). PPAR-γ has been shown to be involved in insulin secretion (Ishida et al., 2004; Kim et al., 2008; Evans-Molina et al., 2009), and it has been suggested that *A. camphorata* might contain PPAR-γ ligands (Suk et al., 2008), so GW9662, a PPAR-γ inhibitor, was used to examine whether PPAR-γ was involved in *A. camphorata*-induced insulin secretion. With co-treatment of 50 μM GW9662 with 250 ng/ml *A. camphorata*, insulin secretion was significantly reduced (**Figure 1B**).

### *Antrodia camphorata* Increased PPAR-γ, GLUT-2, and GLK Expressions in MIN6 Cells

**FIGURE 2:**
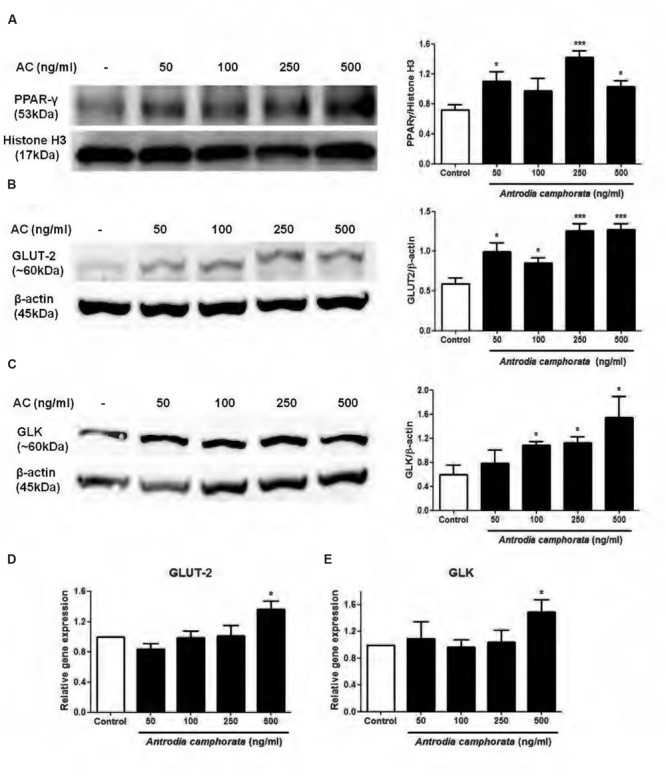
***Antrodia camphorata* increased PPAR-γ expression and its related protein expressions in MIN6 cells.** The cells were treated with *A. camphorata* (50–500 ng/ml) for 24 h. **(A–C)** Immunoblots and representative graphs showing the protein expressions of PPAR-γ, GLUT-2 and GLK, *n* = 4, 5. **(D,E)** Relative gene expressions of GLUT-2 and GLK. The gene expressions were normalised to control, *n* = 4, 5. *^∗^P* < 0.05, *^∗∗∗^P* < 0.001 vs. control. Results were expressed as mean ± SEM.

Since PPAR-γ was involved in the enhancement of insulin secretion by *A. camphorata*, we next examined the nuclear protein expression of PPAR-γ in MIN6 cells. The cells were treated with *A. camphorata* (0–500 ng/ml) for 24 h before protein extraction. 250 and 500 ng/ml *A. camphorata* significantly increased nuclear PPAR-γ protein expressions (**Figure 2A**). Studies have identified PPRE on the promoter regions of GLUT-2 and GLK genes, in which PPAR-γ-RXR complex binds to and upregulates their gene transcriptions (Kim et al., 2000, 2002). So we also examined the protein and mRNA expressions of GLUT-2 and GLK. *A. camphorata* (50–500 ng/ml) significantly increased GLUT-2 protein expression (**Figure 2B**), while it increased GLK protein expression in a dose-dependent manner (**Figure 2C**). Furthermore, 500 ng/ml *A. camphorata* significantly increased GLUT-2 and GLK mRNA expressions (**Figures 2D,E**).

### *Antrodia camphorata* Protected from ER Stress-Induced Apoptosis in MIN6 Cells

**FIGURE 3:**
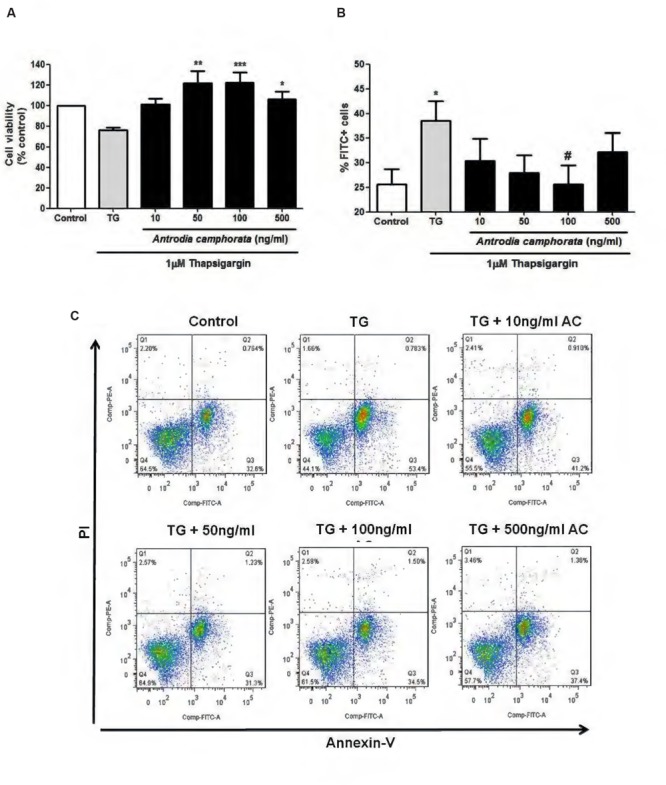
***Antrodia camphorata* reduced ER stress-induced apoptosis in MIN6 cells.** The cells were treated with *A. camphorata* (10–500 ng/ml) for 24 h and apoptosis was induced by 1 μM TG for 24 h. **(A)** MTT assay was performed to measure the cell viability, *n* = 5. *^∗^P* < 0.05, *^∗∗^P* < 0.01, *^∗∗∗^P* < 0.001 vs. TG only. **(B)** Cell apoptosis was measured by Annexin V–PI staining using flow cytometry. Annexin V–fluorescein isothiocyanate (FITC) positive cells were defined as early apoptotic cells, *n* = 7. *^∗^P* < 0.05 vs. control; *^#^P* < 0.05 vs. TG. **(C)** Representative flow cytometric analyses for Annexin V–PI staining. Results were expressed as mean ± SEM.

Endoplasmic reticulum stress is one of the main causes that leads to β-cell apoptosis (Butler et al., 2003; Prentki and Nolan, 2006; Eizirik et al., 2008), so here we used TG as a model to induce ER stress and β-cell apoptosis, and examined the effect of the ethanolic extracts of *A. camphorata* on ER stress-induced apoptosis in MIN6 cells. The cells were treated with *A. camphorata* (10–500 ng/ml) for 24 h and then were stimulated with 1 μM TG for 24 h. MTT assay was performed to determine the cell viability. TG decreased cell viability compared to control, and with pre-treatment of *A. camphorata* (50–500 ng/ml), the cell viability significantly increased (**Figure 3A**). Notably, the increase in cell viability was 47% at 100 ng/ml *A. camphorata*. Annexin V–PI staining was also performed to measure cell apoptosis, and Annexin V–FITC positive cells were defined as early apoptotic cells. Similar results were also demonstrated. TG significantly increased early apoptosis, and *A. camphorata* pre-treatment dramatically suppressed the cell apoptosis (**Figures 3B,C**). 13% reduction in apoptosis was observed at 100 ng/ml *A. camphorata*.

**FIGURE 4:**
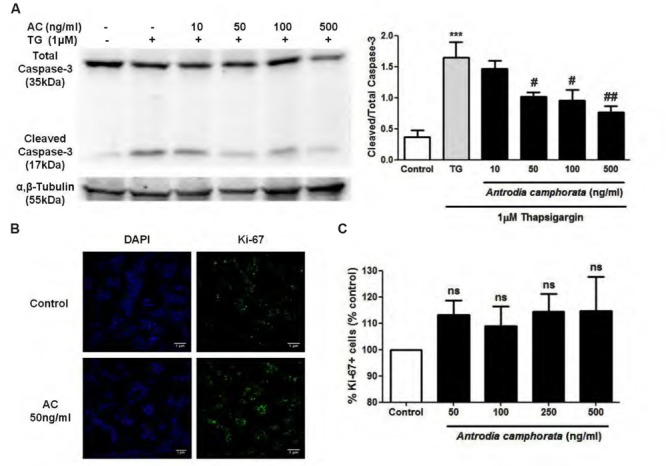
***Antrodia camphorata* decreased caspase-3 expression and slightly increased cell proliferation in MIN6 cells. (A)** Immunoblots and representative graphs showing the protein expressions of cleaved/total caspase-3. The cells were treated with *A. camphorata* (AC; 10–500 ng/ml) for 24 h and then stimulated with 1 μM TG for 24 h, *n* = 4. *^∗∗∗^P* < 0.001 vs. control; *^#^P* < 0.05, *^##^P* < 0.01 vs. TG. **(B,C)** Images and representative graphs showing ki-67 staining. The cells were treated with 50–500 ng/ml *A. camphorata* for 24 h, *n* = 4. Results were expressed as mean ± SEM.

Caspase-3 is another indicator of cell apoptosis, so we next examined the effect of *A. camphorata* on caspase-3 expression by immunoblotting. TG markedly increased cleaved/total capase-3 expression, and *A. camphorata* pre-treatment significantly reduced this expression in a dose-dependent manner (**Figure 4A**). As *A. camphorata* increased cell viability, so cell proliferation was further investigated by ki-67 staining. 50–500 ng/ml *A. camphorata* slightly increased ki-67 staining compared to control (**Figures 4B,C**).

### *Antrodia camphorata* Protected from Apoptosis through Downregulation of IRE1α Pathway in MIN6 Cells

**FIGURE 5:**
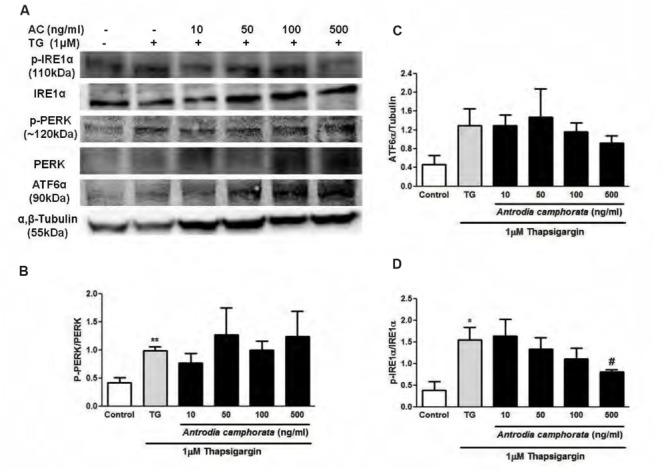
***Antrodia camphorata* reduced ER stress-induced apoptosis by downregulating IRE1α pathway in MIN6 cells.** The cells were treated with *A. camphorata* (AC; 10–500 ng/ml) for 24 h and then stimulated with 1 μM TG for 6 h. **(A)** Immunoblots showing the protein expressions of phospho-IRE1α, IRE1α, phospho-PERK, PERK, and ATF6α. **(B)** Representative graphs showing the protein expressions of phospho-PERK/PERK, *n* = 4. *^∗∗^P* < 0.01 vs. control. **(C)** Representative graphs showing the protein expressions of ATF6α, *n* = 5. **(D)** Representative graphs showing the protein expressions of phospho-IRE1α/IRE1α, *n* = 4. *^∗^P* < 0.05 vs. control; *^#^P* < 0.05 vs. TG. Results were expressed as mean ± SEM.

Thapsigargin is an ER stress inducer, which is known to upregulate UPR responses including IRE1α, PERK, and ATF6α pathways. Next, we investigated whether these pathways were involved in the protection from TG-induced β-cell apoptosis by *A. camphorata*. 6 h treatment with TG increased the protein expressions of phospho-IRE1α/IRE1α, phospho-PERK/PERK, and ATF6α (**Figure 5A**). Pre-treatment of *A. camphorata* (10–50 ng/ml) reduced the increase in phospho-IRE1α/IRE1α expression dose-dependently, but not for phospho-PERK/PERK and ATF6α expressions (**Figures 5A–D**).

## Discussion

*Antrodia camphorata* has been widely used clinically as traditional medicines in many diseases such as hypertension, liver diseases, cancer, and immune-related diseases (Chen et al., 2001; Geethangili and Tzeng, 2011). Previous studies on *A. camphorata* were mostly focused on the anti-cancer, anti-oxidant, anti-inflammatory, and heptaprotective effects (Dai et al., 2003; Hsiao et al., 2003; Mau et al., 2004; Shen et al., 2004; Hsu et al., 2005; Kuo et al., 2006); however, the anti-diabetic effect has never been studied. T2DM is a metabolic disease which is characterized by β-cell dysfunction and apoptosis, and it affects more than 2% of the world population (Day, 2001). Therefore, we here were the first to examine the effects of the ethanolic extracts of *A. camphorata* on insulin secretion and apoptosis in a mouse β-cell line, MIN6, and the underlying mechanisms.

In the present study, we first demonstrated that *A. camphorata* increased glucose-induced insulin secretion dose-dependently in MIN6 cells. At 500 ng/ml *A. camphorata* with 16.7 mM glucose KRBB stimulation, the increase in insulin secretion was fivefold higher than control. Rosglitazone, a PPAR-γ agonist, was used as a positive control, and its insulin secretion was only 1.2-fold higher than control, therefore it suggested that *A. camphorata* might have anti-diabetic effect. Moreover, at basal glucose level (5.5 mM glucose), 250 and 500 ng/ml *A. camphorata* also significantly increased insulin secretion, this suggested that *A. camphorata*-induced insulin secretion was dispensable for glucose response. Furthermore, it has been suggested that *A. camphorata* might contain PPAR-γ ligands (Suk et al., 2008), so PPAR-γ inhibitor, GW9662, was used to investigate whether PPAR-γ was involved in the enhancement of insulin secretion by *A. camphorata*. GW9662 markedly reduced the enhancement of insulin secretion by *A. camphorata*, so this suggested that *A. camphorata* enhanced insulin secretion through PPAR-γ pathway. PPAR-γ is a transcription factor which is located in the nuclear membrane, and it forms heterodimers with RXR to bind to the PPRE region on promoters and up-regulates gene transcription (Kim and Ahn, 2004). Activation of PPAR-γ has been shown to increase intracellular calcium concentration, which leads to insulin release (Kim et al., 2008, 2013). It has been shown that GLUT-2 and GLK promoters contain PPRE region, so PPAR-γ up-regulates GLUT-2 and GLK gene transcription (Kim et al., 2000, 2002). Therefore the expressions of PPAR-γ, GLUT-2 and GLK were also examined. GLUT-2 is a glucose transporter which helps to uptake glucose into the cells, while GLK is an enzyme which facilitates the phosphorylation of glucose inside the cells. Our results showed that *A. camphorata* increased nuclear PPAR-γ protein expressions, GLUT-2 and GLK protein and mRNA expressions in MIN6 cells. Taken together, this suggested that *A. camphorata* increased insulin secretion through PPAR-γ pathway, and upregulated PPAR-γ, GLUT-2 and GLK expressions in MIN6 cells.

Endoplasmic reticulum stress is one of the main causes in T2DM, which leads to β-cell apoptosis and dysfunction (Butler et al., 2003; Prentki and Nolan, 2006; Eizirik et al., 2008). Cell apoptosis in β-cells is one of the main concerns in T2DM, and it has been demonstrated that *A. camphorata* prevented hepatic cell damage by serving as radical scavengers (Hsiao et al., 2003; Song and Yen, 2003), and ameliorated liver damage in animal model of nonalcoholic liver disease with high-fat-diet (Chou et al., 2013). However, it has not been studied in β-cell death, so we further investigated the effect of *A. camphorata* on ER stress-induced cell death in MIN6 cells. TG was used as a model to induce ER stress, thus β-cell apoptosis. Our results demonstrated that *A. camphorata* pre-treatment significantly improved the cell viability and reduced TG-induced early apoptosis. This suggested that *A. camphorata* could prevent from ER stress-induced cell death. In addition, *A. camphorata* significantly reduced cleaved/total caspase-3 expression, another marker of cell apoptosis. Taken together, our findings suggested that *A. camphorata* has a protective role against ER stress-induced apoptosis in MIN6 cells. β-cell dysfunction is one of the main outcomes in T2DM, and beside from treating T2DM by reducing β-cell apoptosis, β-cell regeneration also plays an important role for the treatment of T2DM (Dominguez-Bendala et al., 2012; Minami and Seino, 2013). Ki-67 is a nuclear protein which is present in all phases of cell cycle except resting cells, so it is associated with cell proliferation (Scholzen and Gerdes, 2000). In addition, ki-67 staining is used commonly as a cell proliferation marker. Our results demonstrated that *A. camphorata* slightly increased cell proliferation in MIN6 cells, so this suggested that *A. camphorata* might be potential to enhance cell regeneration in MIN6 cells. Furthermore, a study showed that the plasma levels of glucose, leptin, insulin, total cholesterol, and triglyceride were lowered in high-fat diet mice treated with Ergostatrien-3β-ol from *A. camphorata*, and insulin resistance was also attenuated in these mice (Kuo et al., 2015). Taken together, our study together with their study suggested that *A. camphorata* might have anti-diabetic effects and might be potential for treating T2DM.

In addition, we also examined the downstream pathways that were involved in β-cell protection by *A. camphorata*. During ER stress, misfolded proteins accumulate in the ER lumen, which activate UPR and prevents the accumulation of misfolded proteins (Leibowitz et al., 2010), and UPR is regulated by IRE1α, PERK, and ATF6α pathways (Leibowitz et al., 2010; Back et al., 2012). IRE1α is a ER transmembrane kinase, and during ER stress, it undergoes autophosphorylation which induces endoribonuclease activity and splices XBP-1 mRNA that regulates chaperone expressions (Fonseca et al., 2011). In addition, prolonged activation of IRE1α by high glucose induced β-cell death (Hou et al., 2008). Herein we showed that 6 h treatment with TG significantly increased IRE1α, PERK, and ATF6α pathways. With TG stimulation for 6 h, pre-treatment with *A. camphorata* reduced the protein expression of phospho-IRE1α/IRE1α in a dose-dependent manner, but not for phospho-PERK/PERK and ATF6α expressions. This suggested that *A. camphorata* could protect the cells as early as 6 h by downregulating IRE1α pathway. These findings were consistent with other studies suggesting that reducing ER stress could prevent β-cell death (Song et al., 2008; Zhu et al., 2013).

## Conclusion

The present study demonstrated, for the first time, the potentiating effect of *A. camphorata* on insulin secretion and the protective effect against ER stress-induced apoptosis in a mouse β-cell line, MIN6. We demonstrated that *A. camphorata* potentiated glucose-induced insulin secretion dramatically through PPAR-γ pathway, and upregulated genes which were involved in insulin secretion, including PPAR-γ, GLUT-2, and GLK. We also provided evidences that it has a protective effect against TG-induced cell apoptosis. In addition, *A. camphorata* was able to slightly increase cell proliferation in MIN6 cells. Taken together, our findings suggested that *A. camphorata* has beneficial effects on improving MIN6 cell function, so it might have anti-diabetic effects and could be a novel drug for T2DM.

## Author Contributions

CV, HT, YK, SL, and MH designed the study, developed the methodology, performed the data analysis and approved the final version of the manuscript. CV and HT performed the experiments. CV wrote the manuscript.

## Conflict of Interest Statement

The authors declare that the research was conducted in the absence of any commercial or financial relationships that could be construed as a potential conflict of interest.

The reviewer AD-V and handling Editor declared their shared affiliation, and the handling Editor states that the process nevertheless met the standards of a fair and objective review.

## Abbreviations

*A. camphorata*: *Antrodia camphorata*
DMEM: Dulbecco’s Modified Eagle’s Medium
ER: endoplasmic reticulum
GLK: glucokinase
GLUT-2: glucose transporter-2
IRE1α: inositol requiring 1α
MTT: thiazolyl blue tetrazolium blue
PI: propidium iodide
PPAR-γ: peroxisome proliferator-activated receptor-γ
PPRE: peroxisomal proliferator response element
RXR: retinoid X receptor
T2DM: type 2 diabetes mellitus
TG: thapsigargin
UPR: unfolded protein response
XBP-1: X-box binding protein 1
